# Meeting in the Middle: Towards Successful Multidisciplinary Bioimage Analysis Collaboration

**DOI:** 10.3389/fbinf.2022.889755

**Published:** 2022-04-14

**Authors:** Anjalie Schlaeppi, Wilson Adams, Robert Haase, Jan Huisken, Ryan B. MacDonald, Kevin W. Eliceiri, Elisabeth C. Kugler

**Affiliations:** ^1^ Morgridge Institute for Research, Madison, WI, United States; ^2^ BioImaging and Optics Platform, École Polytechnique Fédérale de Lausanne (EPFL), Lausanne, Switzerland; ^3^ Department of Biomedical Engineering, Vanderbilt University, Nashville, TN, United States; ^4^ Department of Pharmacology, Vanderbilt University, Nashville, TN, United States; ^5^ DFG Cluster of Excellence “Physics of Life”, Germany and Center for Systems Biology Dresden, TU Dresden, Dresden, Germany; ^6^ Department of Biology and Psychology, Georg-August-University Göttingen, Göttingen, Germany; ^7^ Faculty of Brain Sciences, Institute of Ophthalmology, University College London, London, United Kingdom; ^8^ Center for Quantitative Cell Imaging, University of Wisconsin-Madison, Madison, WI, United States

**Keywords:** computational science, bioimage analysis, collaboration, guide, multidisciplinary

## Abstract

With an increase in subject knowledge expertise required to solve specific biological questions, experts from different fields need to collaborate to address increasingly complex issues. To successfully collaborate, everyone involved in the collaboration must take steps to *“meet in the middle.”* We thus present a guide on truly cross-disciplinary work using bioimage analysis as a showcase, where it is required that the expertise of biologists, microscopists, data analysts, clinicians, engineers, and physicists meet. We discuss considerations and best practices from the perspective of both users and technology developers, while offering suggestions for working together productively and how this can be supported by institutes and funders. Although this guide uses bioimage analysis as an example, the guiding principles of these perspectives are widely applicable to other cross-disciplinary work.

## Introduction

Existing perspectives on multidisciplinary scientific collaborations are largely written from the perspective of one domain, lacking the insights into the range of fields required to collaborate on given problems. This is particularly seen in bioimage analysis where diverse expertise, including that of biologists, microscopists, data analysts, clinicians, engineers, and physicists, intersect. Here, we suggest steps towards successful collaboration in bioimage analysis and beyond by “*meeting in the middle.*” Even though we present collaborations between biologists and computer scientists in biomedical image analysis, most of our recommendations apply to other multidisciplinary collaborations.

Advancements in modern imaging modalities have led to achieving greater spatial and temporal resolution. As a result, modern imaging approaches produce large datasets up to petabytes in size. To deal with this data load there is an accompanying need for computational analysis that facilitates science to move from subjective visual assessment to objective quantification. However, modern imaging data size and complexity, together with the need for flexible analysis routines, produce “bottlenecks” between processing data to retrieving biological insights. Particularly with biomedical image analysis, customized analysis requires expertise integrated from different fields to produce reproducible and effective tools (Sandve et al., 2013; Osborne et al., 2014). However, interdisciplinary work requires considerable effort from researchers to acknowledge their collaborators’ practices (Box 1); as well as considerations across fields, institutes, lab groups, funders, and stakeholders. Even when tools are developed by interdisciplinary collaborations, disconnects between data analysts and experimental scientists may lead to tool underutilization, such as: scientists may be unaware of tools, unable to use them, not understand the importance of a particular technical parameter, or inability to adapt a tool to their research questions (Tröger et al., 2020). In this perspective, from a group of biological end-users and computational analysts, we examine the use case of bioimage analysis, discussing considerations and best practices.

BOX 1 **Practices may differ between fields**

Open discussions are needed to acknowledge the potential differences in practices between scientific fields. Three examples include:

a) **Publication timelines**: Appreciating the timelines of others is essential. E.g., Does the experiment take days or months? How long does it take to develop, test, and validate code? How adaptable is the analysis? Computational fields generally prioritize sharing novel aspects of their work in annual conference proceedings, with open-source projects often being published in scientific journals years after initial release and community adoption. Contrarily, biological fields often do not release insights, data, or procedures before scientific publication.
b) **Authorship order**: In mathematical sciences, alphabetical authorship is often applied, while biological sciences associate first authorship with principal contributors and last authorship with principal investigators.
c) **Research data policies**: Different countries, institutes, fields, and funders apply distinct policies for research data (e.g., sharing, storage, anonymization, archiving, copyright).

## Steps That all Scientists and the Community can Take

### Clarification of Expectations and Aims

Successful collaboration begins with communication and clarifying expectations. Particularly in early meetings, communication is key as scientists from different fields need to find a common language and understand that words often have different meanings in different fields. For example, in biology “model” refers to an organism; whereas, in computer science, it typically refers to mathematical equations.

Successful collaborations are win-win situations; investing in understanding your collaborators’ interests facilitates a fruitful outcome (Box 2). Collaboration is also an iterative process, benefiting from regular clarification of priorities and expectations. It is important early on to understand and set expectations for the project and discuss with collaborators how this project fits into their current and long-term goals. Openness and non-judgment are paramount for successful collaborations, with clear communication being essential for each step. It is also important to discuss how the collaborative work itself is conducted.

BOX 2 **Collaboration is a two-way street**

Successful collaborations not only involve a common interest in the project but also between the collaborators. Getting to know your collaborators facilitates effective and successful completion of the project. Consider the motivations of collaborators and yourself with regards to authorship, financial support, or other compensations:

a) **Involvement and responsibilities**: Understanding expectations helps to anticipate the level of involvement and resources of others. E.g., Does the life scientist want to be involved and taught how to perform the analysis, or do they want to outsource the analysis?
b) **Compensation**: While in academic collaborations often a “quid pro quo” compensation, such as authorship or acknowledgment, is assumed, financial compensation should be openly discussed. When outsourcing experiments or data analysis it is important to appreciate that this is a service. Thus, financial compensation, authorship, and acknowledgements require clarification. The provision of services with financial compensation does not equal a rejection of authorship, particularly when providing substantial contributions, critical intellectual content and accountability for parts of the project. The specifics of this will need to be discussed and depend on contracts and journal guidelines. For example, Contributor Roles Taxonomy (CRediT) can be used as starting point to discuss authorship versus contributorship (Allen et al., 2019).

### Sharing Tools and Data

One essential consideration is to share tools and data under open-source and open-access licenses. Not only is this important due to public funding of academic research, but studies with open-access, available images, and code are most repeatable and reproducible. A 2018 study examining computational reproducibility showed that out of 204 preprint publications only 44% shared code or data, and only 26% could be reproduced (Serghiou and Ioannidis, 2018). Furthermore, research published under findable, accessible, interoperable, and reusable (FAIR) guidelines (MacLeod, 2018) has high impact (Vandewalle, 2012); similarly, research preprints increase research impact (Serghiou and Ioannidis, 2018). Sharing code and analysis workflows is increasingly facilitated by data repositories such as Zenodo (Zenodo - Research., 2021), EBI (EMBL-EBI, 2021), or IDR (IDR: Image Data Resource, 2021). Publishers support this forward-facing approach, for example, eLife’s Executable Research Article initiative allows more transparency, reproducibility, and interactivity with published articles (Tsang and Maciocci, 2020). Sharing data, metadata, code, and documentation are often perceived as additional work that relies on individuals. Future directions could include support from institutes or funders on “data best practice,” *i.e.,* white papers on research data and tool management are widely lacking. This can establish data management and sharing as a part of research routine. Open data assures that the developers and data analysts can continue or start to work with the data beyond the direct project for which these data were produced. Similarly, sharing of code and data analysis approaches allows for the reuse and sharing of the code by collaborators and new adopters.

### Communication

In addition to sharing data, code, and tools, goals need to be clearly communicated and shared by all fields involved. By communicating the concepts behind a tool, more people will know about it and are likely to use it. Similarly, having biologists explain what value a tool brings to current medical or societal questions opens multi-directional engagement. Traditionally, this is achieved by conference presentations, websites, or redirecting interested users to common directories (e.g., GitHub). However, with ever-evolving communication platforms, science communication is also available via YouTube, dedicated wiki platforms, and social media, like Twitter or TikTok (Habibi and Salim, 2021). As marketing and dissemination are time-consuming, it cannot fall solely into the responsibility of developers, but also end-users of the tool. For example, recording a “how-to” tutorial or programming a workflow for a targeted use case leverages the experience, wisdom, and resources of the biomedical imaging and image analysis community can be extremely impactful. Importantly, even though dedicated positions for science communicators begin to emerge, they are still rare at institutions. Thus, people who engage in science communication often do this in addition to and outside of their original job descriptions. This is surprising, considering that pharma companies often invest more into marketing than actual research (Lopez, 2015). So why not use these concepts of communication, marketing, and strategy in science to raise awareness about results and initiate collaborations?

## Challenges to be Considered

### Bridge Scientists

To facilitate communication and productive multidisciplinary projects, scientists that understand the different fields involved and who “speak each language” are required. Such *“bridge scientists”* often start their careers in one field and transition across disciplines. For example, biologists working in biomedical image analysis start developing code to advance their projects, or physicists perform experimental wet lab work to prove their theoretical models. Positions to train and fund such scientific liaison roles are growing in availability but institutes and funders still struggle to keep pace with modern research demands, and responsibilities. Similarly, it is often assumed to be the bridge scientist’s responsibility to provide user-friendly tools. But most scientists’ goal is to innovate, develop, or discover, rather than building application support for end-users, such as writing user-friendly interfaces for biologists or building user-friendly microscopes.

Cross-disciplinary collaborations often require equal contributions from multiple scientists, which result in co-authorship. However, scientists who programmed the data analysis routines are often not first authors in biological research publications but are listed in middle-author positions as they are often considered support scientists. Having said that, the academic system to measure impact (e.g., when applying for grants and positions) does not always value middle authorship despite their crucial input. We suggest a mindset change on how middle-authors are regarded, e.g., this can be supported by emphasizing their individual roles using the CRediT (Allen et al., 2019) more prominently to highlight researcher specialization. Another suggestion is could be to use partial alphabetical authorship, meaning only the primary and last author are defined by their role, while other are defined by name (Mongeon et al., 2017). This important issue should be discussed throughout interdisciplinary approaches with author orders and co-positions being considered as appropriate. The use of co-first and co-senior authors can be an effective solution when there is more than one significant contributor.

### Funding and Community Efforts

Another challenge is encountered when a biomedical image analysis manuscript is reviewed by specialists in biology and computer science; as both experts are likely to aim for more in-depth contributions to their respective fields, missing the multidisciplinary novelty. This lack of cross-disciplinary journals and review mechanisms often increases pressure on bridge scientists, particularly early-career researchers who need publications for career progression.

Moreover, when early-career bridge scientists progress, the resources to maintain, adapt, teach, and distribute the developed tools are often lacking. Hence, many tools are abandoned. Lack of long-term support for projects from institutes and funders makes them extinct and other scientists find themselves reinventing past solutions, wasting valuable resources.

Funding sources have begun to recognize the importance of interdisciplinary bridge scientists in developing open-source tools. Examples of sustainably funded bioimaging software include such ImageJ/Fiji (Schindelin et al., 2012; Schneider et al., 2012; Rueden et al., 2017), CellProfiler (Jones et al., 2008), scikit-image (Van Der Walt et al., 2014), NumPy (Harris et al., 2020), Napari (Sofroniew et al., 2021), and DeepLabCut (Mathis et al., 2018). There are also crucial efforts driven by the community to help bridge the gap between experimentalists and developers. Online platforms such as the Image Science Forum image.sc (Rueden et al., 2019), micro-forum (Microforum - Light microscopy forum, 2021), and the NEUBIAS Academy YouTube channel (NEUBIAS - YouTube, 2021). However, these communities are only successful if scientists from different fields contribute and if sufficient funding is available.

## The Steps Experimental Scientists can Take

In addition to the more community-oriented issues discussed above and the need to clarify, there are specific steps the imaging/experimental scientist can take to foster successful collaborations. Data analysis collaborations typically start with experimental design and data acquisition by experimentalists, here, biologists. However, rather than directly starting with experiments, we support the idea of reverse experimental design, where collaboration starts before the experiment (Vahey and Fletcher, 2014) (Box 3).

BOX 3 **Be prepared**

Because experimental scientists are the ones posing the biological question to answer, the larger their involvement in data analysis, the more meaningful the outcomes.

a) **Explaining scientific questions**: When experimental scientists approach data analysts, it is critical to not just send data, but spend time explaining the scientific question, data, its acquisition, and desired answers. The more collaborators know about each other’s work, the more accurate analysis workflows are produced.
b) **Knowing the parameters**: As data analysis is highly complex, data scientists cannot explain all minute details and parameters that could be analyzed (e.g., intensity/spatial dynamics, object numbers, lineage tracking). Thus, experimental scientists could examine current literature for parameters to answer their biological question, e.g., Is treatment likely to reduce vascular growth, and, if yes, could this be quantified by vessel numbers or branching points?
c) **Iterations**: Particularly in imaging, the analysis strongly informs data acquisition. It is important to put the software tools in the tester’s hand as soon as possible. This approach known as “rapid prototyping” or “release-early-release-often” promotes sharing software in early stages, to collect feedback to increase the chance of achieving the software goal (Raymond, 1999). Once an initial data examination and analysis are done, often improvements in sample preparation and/or data acquisition are suggested.
d) **Examples:** Having a dataset or an end-result example at hand to discuss greatly facilitates the understanding and interaction as it shifts the conversation from passive to more active. Example data should be published together with the analysis code to enable others to adopt the tool for processing their data.

Together, experimental scientists should initiate communication with data analysts before data acquisition, and iterative feedback facilitates meaningful and most effective collaboration.

## The Steps Computational Scientists can Take

Similar to experimental scientists who need to explain data and what they would like to quantify, computational scientists need to communicate what can be reliably quantified and indicate whether provided image data is suitable for a certain kind of analysis or if the experimental design requires changes (Wait et al., 2020). Data analysts should also understand that repeating an experiment to improve image data quality can come at high financial cost and brings its own challenges. Shadowing experimental scientists to learn how experiments are done and images are taken, can be very insightful. However, as this again goes beyond typical job descriptions, there needs to be the space and support from institutes and funders to do so. Raising awareness of the efforts undertaken to acquire data can lead to novel approaches, better images, and improved results.

### Installation

Contrary to serving colleagues with custom image analysis workflows, tool developers aim at a large target audience. Thus, experimental scientists may only hear back from the computational scientist after a longer time. We recommend tool developers talk to their collaborators frequently to make sure developed tools fit the need and to learn from the experimentalists about adjacent or future questions. In that way, it is possible to design tools useful for a broad audience. Furthermore, tool adoption highly depends on the ease-of-use. Intuitive and user-friendly tools have better chances to be used, and importantly, to be used correctly. The first barrier to using a tool is installation*.* We observe colleagues in the life sciences struggling with command-line-based installation instructions, limitations regarding the operating system, and dependency management (Levet et al., 2021). One of the reasons why Fiji (Schindelin et al., 2012) is so successful in the life sciences is because its updater simplifies the installation of plugins so that hardly any technical skills are necessary. Plugins from other scientists simply run after activating their update site (Haase, 2021). Remotely accessible software, prepackaged virtual machines, and cloud computing may bring new convenient solutions circumventing complicated installation instructions in the future. But again, there is a need for support from institutes and funders, not only in form of financial support but also by providing infrastructure, service roles, and maintenance.

### Licenses

Another critical aspect in cross-disciplinary collaborations is licensing, and the reasons why software is licensed in a certain way are manifold. However, we urge developers to publish under open-science licenses. Furthermore, we motivate image analysis workflow script developers to clearly state the license of their code, e.g., in a header section of the script file. Licensing is pivotal even if a custom script was written for an individual user to clarify things such as copyright, use, adaptation, and publication rights.

### Usability

In terms of usability, graphical user interface (GUI) or application programming interface (API) facilitate uptake. Similarly, configuration dialogs with reasonable default parameters and helpful tool-tips are appreciated by end-users, and keeping advanced parameters hidden in “advanced” tabs supports usability. Creating user interfaces that fit well to the end-users needs is an iterative time-consuming process. Different users see interfaces differently, and typically end-users have a different understanding of data, algorithms, and parameters than developers. Continuous discussion, regular meetings, and a short software release cycle facilitate making user-friendly GUIs.

### Documentation

Documentation of the developed tool is pivotal, and where possible should be built-in. This includes technical documentation for developers, explanations of requirements for IT departments, and external use-oriented descriptions for end-user (Sofroniew et al., 2021). For *IT departments*, requirements such as system configuration, rights, and system architecture could be documented in a wiki or website, making information accessible without distracting end-users. For *developers*, the most essential documentation is code comments, including general explanations and specific details. For *users*, step-by-step guides can be created, like experiment lab protocols. We recommend providing walk-through tutorials of the software together with example data. For example, start with what an experiment might look like, and work backward to what the user needs to do in each processing step. This is supported by using more interactive ways to present workflows, commentary, and images, such as well-developed websites, Python Jupyter Notebooks (https://jupyterbook.org/), or MatLab Live Scripts (https://uk.mathworks.com/help/matlab/live-scripts-and-functions.html). See some examples: https://imagej.net/, https://scikit-image.org/, https://cupy.dev/, https://qupath.github.io/, https://clij.github.io/, https://gitlab.com/gernerlab/cytomap/-/wikis/Home https://github.com/stardist/stardist, https://github.com/spacetx/starfish, https://github.com/OakesLab/AFT-Alignment_by_Fourier_Transform, https://github.com/WhoIsJack/data-driven-analysis-lateralline, https://haesleinhuepf.github.io/BioImageAnalysisNotebooks/.

Additionally, it is useful to document the software purpose, along with indications for what input data variability can be tolerated and how this impacts the result. Furthermore, giving concise explanations of parameters and how they can be tweaked from experiment-to-experiment for optimal performance and different use cases is crucial. Screen recordings from training sessions make for great online tutorials. However, the more in-built and intuitive documentation, the easier. A help button next to an input field explaining the parameter is preferred over internet search for help. Another step is to provide an example data set, code, and documentation of expected results. Lastly, Frequently Asked Questions (FAQ) with common mistakes and issues can be compiled alongside tool development.

### Tool Maintenance

Tool maintenance is often a critical challenge. The earlier plans are made for tool management and maintenance the better (e.g., tool support, tool updates, costs, individual responsibilities). Our experience shows that many tools developed in cross-disciplinary collaborations are built by early career researchers, who then naturally progress away from a project when moving forward in their careers. Thus, tool documentation, dissemination, and maintenance often depend on financially non-compensated altruism or tools fall into despair.

Together, considering steps to reduce barriers of tool-use in installation, licensing, usability, documentation, and maintenance is pivotal, with communication across disciplines again being essential for successful collaboration.

## Discussion

Examining aspects for successful multidisciplinary research, clear, and open communication is a common thread. All parties of cross-disciplinary projects need to engage beyond their typical comfort zone to learn on personal (e.g., expectations), practical (e.g., authorship), and interpersonal (e.g., terminology) levels. Here, we provide suggestions to foster such collaborations at the personal, professional, public, and institutional levels in hopes that the growing field of bioimage analysis can benefit all aspects of scientific research. Patience, openness, and sympathy between all parties involved go a long way in ensuring collaboration is successful. A shared desire to learn while helping advance each other’s research ensures that collaboration is mutually beneficial for all involved while yielding impactful tools for the broader scientific community. Further, data, tools, and documentation must be openly shared to avoid resources falling into dilapidation. New funding, positions, and careers are needed to make space for collaborative work to push boundaries that individual fields might not be able to achieve. Even though it may sound intuitive, communication is most often the biggest challenge towards collaborators truly meeting in the middle.

We strongly urge the support and creation of positions and career tracks for “bridge scientists”; and the normalization of co-authors and co-corresponding authors with equal contribution to be truly considered as co-positions. This includes a change of mindset across scientific fields, that bridge scientists deliver expertise in cross-communication and work beyond their field of expertise.

Collaborations between biologically and computationally focused researchers have the potential to push the frontiers of biomedical research forward. While unquestionably promising and valuable, facilitating such collaborations takes a collective sustained effort. Bridge scientists can help foster such projects and partnerships, but funding and institutional support for such roles need to be strengthened. As experimental imaging capabilities continue to outpace our ability to store, process, and interpret these data, the information provided here should help in establishing the needed interdisciplinary collaborations to effectively address opportunities and challenges presented by modern research data.

## Data Availability

The original contributions presented in the study are included in the article/Supplementary Material, further inquiries can be directed to the corresponding authors.
